# Lime and ammonium carbonate fumigation coupled with bio‐organic fertilizer application steered banana rhizosphere to assemble a unique microbiome against Panama disease

**DOI:** 10.1111/1751-7915.13391

**Published:** 2019-03-05

**Authors:** Zongzhuan Shen, Beibei Wang, Jiaxin Zhu, Hangwei Hu, Chengyuan Tao, Yannan Ou, Xuhui Deng, Ning Ling, Rong Li, Qirong Shen

**Affiliations:** ^1^ Jiangsu Provincial Key Lab of Solid Organic Waste Utilization Jiangsu Collaborative Innovation Center of Solid Organic Wastes Educational Ministry Engineering Center of Resource‐saving Fertilizers Nanjing Agricultural University Nanjing 210095 Jiangsu China; ^2^ Hainan Key Laboratory for Sustainable Utilization of Tropical Bio‐resources Institute of Tropical Agriculture and Forestry Hainan University 570228 Haikou China; ^3^ Faculty of Veterinary and Agricultural Sciences University of Melbourne Melbourne Vic. 3010 Australia; ^4^ Ecology and Biodiversity Group Department of Biology Institute of Environmental Biology Utrecht University Utrecht The Netherlands

## Abstract

Microbiome plays a key role in determining soil suppressiveness against invading pathogens. Our previous study revealed that microbial community of bulk soil could be manipulated by lime and ammonium bicarbonate fumigation followed by biofertilizer application. However, the assembly of microbial community suppressive to banana Panama disease in the rhizosphere is still unclear. In this study, we used high‐throughput sequencing and quantitative PCR to explore the assembly of rhizosphere microbiome associated with banana Panama disease suppression in a two‐seasonal pot experiment. We found biofertilizer applied to lime and ammonium bicarbonate fumigated soil significantly (*P *< 0.05) reduced the abundance of rhizosphere *Fusarium oxysporum* compared to biofertilizer applied to non‐fumigated soil. Principal coordinate analysis revealed that biofertilizer applied to lime and ammonium bicarbonate fumigated soil re‐shaped the rhizosphere bacterial community composition by increasing the phylogenetic relatedness, and stimulating indigenous microbes, *for example*,* Gemmatimonas*,* Sphingomonas*,* Pseudomonas*,* Lysobacter* and *Bacillus*. Co‐occurrence analysis revealed that potential species involved in disease suppression were more interrelated in disease‐suppressive soils. Taken together, lime and ammonium bicarbonate fumigation followed by biofertilizer application could induce banana rhizosphere to assemble beneficial microbes dominated consortia to suppress banana Panama disease.

## Introduction

Interactions between microbes in bulk and rhizosphere soil, and plant provide a potential to dynamically affect plant health and production through complex feedback mechanisms (Chaparro *et al*., 2012). Since rhizosphere microbiota acts as the first line of plant defence against soil‐borne pathogens (Cook *et al*., 1995; Philippot *et al*., 2013), the study of how plant recruits beneficial microbes from bulk soil to assemble a disease‐suppressive microbiome in the rhizosphere could provide an important understanding of plant–soil–microbe interactions associated with soil‐borne disease suppression.

Rhizosphere, known as the narrow region of soil that adheres to plant roots and influenced by plant exudates, soil type and agricultural practices (Haichar *et al*., 2008; Berg and Smalla, 2009; Lundberg *et al*., 2012), is an environment where the beneficial and pathogenic microorganisms exert significant influences on plant health and growth (Bais *et al*., 2006; Philippot *et al*., 2013). Given that bacteria is the most diverse and abundant group of microorganisms inhabited in the rhizosphere and most antagonistic to *Fusarium* pathogen (Raaijmakers *et al*., 2009), bacterial community in rhizosphere plays a key role in managing soil‐borne diseases. Therefore, it is essential to figure out how roots assemble a disease‐suppressive bacterial community in rhizosphere to support sustainable crop production.

It has been widely recognized that microbial community assembly is driven by both deterministic and stochastic processes (Stegen *et al*., 2012; Evans and Wallenstein, 2014; Dini‐Andreote *et al*., 2015). Deterministic process is defined as the determination of identity and abundance of species by environmental filtering or species interaction (Tilman, 2004; Chase, 2007), while the stochastic process known as microbial community dynamics is the sum of individual stochastic events, *that is*, drift, dispersion and speciation (Nemergut *et al*., 2013; Stegen *et al*., 2013). Due to the relatedness between the phylogenetic distance of species and their ecological similarity, phylogenetic community structure combined with the ecological null model can be used to infer the relative contributions of deterministic and stochastic processes to microbial community structure (Webb, 2000; Webb *et al*., 2003). In general, when microbial community is driven by deterministic processes, the phylogenetic structure is clustered or over dispersed while the phylogenetic community structure is not significantly different from the null model when microbial community is driven by stochastic processes. However, the phylogenetic response and ecological process driving the microbial community assembly in banana rhizosphere influenced by agricultural management practices remains largely unknown (Vellend, 2010; Kembel *et al*., 2011).

Banana is the most popular fruit in the world and an important source of nutrition for people (FAOSTAT, 2017). Virtually more than half of the known banana cultivars belong to the *Cavendish* subgroup of the species, which are genetically nearly identical (Ploetz, 2015). The worldwide monoculturing of genetically identical plants makes the *Cavendish* banana cultivation highly vulnerable to disease outbreaks. One of the most prominent examples is the Panama disease, a soil‐borne disease caused by the fungus *Fusarium oxysporum* f. sp. *cubense* race 4 (*Foc*4), which can infect the root and vascular system of plants and persist in the soil for decades (Ploetz, 2006). The wilt disease is a significant biotic limitation to sustainable production of the *Cavendish* banana, especially in China, which is the second largest banana producer in the world (Butler, 2013; Ordonez *et al*., 2015).

Panama disease of banana is difficult to control due to the survival of *Foc*4 as chlamydospores in soil or as a saprophyte of non‐host plants (Deltour *et al*., 2017). In our previous study, application of a biofertilizer produced by fermentation of compost and amino acids inoculated with a *Bacillus* sp. biocontrol agent into the *Foc*4 invaded soil, effectively controlled the Panama disease under banana mono‐cropping conditions (Shen *et al*., 2015). The controlling effect was more pronounced when the biofertilizer‐amended soil was previously treated by fumigation, which partly attributed to soil microbiome manipulation towards a disease‐suppressive microbial community. Microbial community composition in the bulk soil treated with fumigant and biofertilizer has been previously reported to be associated with disease suppression (Shen *et al*., 2018). However, how this manipulated microbiota in bulk soil influences the assemblage of microflora in banana rhizosphere is partially known. Furthermore, the correlations between this assembled rhizosphere microbiome and banana health are also unexplored.

Considering the changes in soil properties and reduced microbial biomass and diversity during fumigation as previously reported (Shen *et al*., 2018), we hypothesized that soil microbial community is re‐shaped by fumigation and then re‐assembled after amendment of disease‐suppressive compost, resulting in banana microbiome that can suppress Panama disease. To test this hypothesis, a two‐season pot experiment was performed to investigate how the bacterial community was assembled in the banana rhizosphere when biofertilizer was applied to a soil fumigated by lime and ammonium bicarbonate. The potential disease‐suppressive mechanisms were also explored at the whole microbiome level and the specific indigenous microbes associated with disease suppression were identified.

## Results

### Abundance of *F. oxysporum* in banana rhizosphere

**Figure 1:**
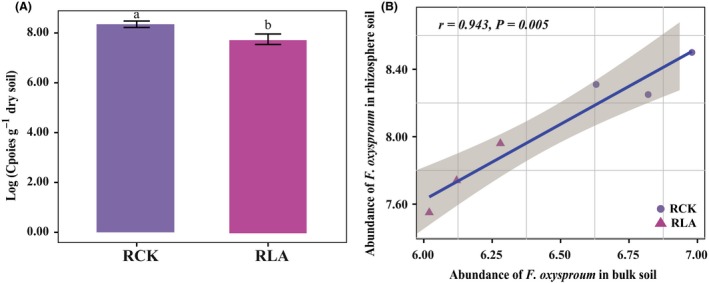
Histogram of log‐transformed abundance of *F. oxysporum* in the rhizosphere soils quantified by the qPCR analysis (A). Pearson correlations between abundance of *F. oxysproum* in bulk soil and abundance of *F. oxysproum* in rhizosphere soil (B). Bars above the histogram represent standard deviation and different letters indicate a significant difference (*P *< 0.05) according to two independent sample *t*‐test. RCK and RLA represent rhizosphere soil sample collected at the end of pot experiment of the LAB treatment and CKB control respectively. CKB, treatment of biofertilizer applied to non‐fumigated control; LAB, treatment of biofertilizer applied to mixture of lime and ammonium bicarbonate fumigated soil.

Among the soils sampled at the end of pot experiment during the second season, the treatment of biofertilizer applied to lime and ammonium carbonate fumigated soil (LAB) with lower disease incidence showed a significantly lower abundance of *F. oxysporum* in the rhizosphere soils than that in the treatment of biofertilizer applied to non‐fumigated soil (CKB) (Fig. 1A). The abundance of *F. oxysporum* in the rhizosphere soils displayed a significant and positive correlation with that in the bulk soils (Fig. 1B).

### Overview of the sequencing data

In total, 386 323 high‐quality sequences for 16S rRNA genes were retained after quality filtering and removal of archaeal sequences (Table S1). Based on the 97% similarity, a total of 5488 OTUs were obtained for the bacterial 16S rRNA genes across all the samples. The most five abundant phyla Proteobacteria, Bacteroidetes, Firmicutes, Acidobacteria and Actinobacteria accounted for 78.9% of the total bacterial sequences (Fig. S2). The bacterial community composition was significantly correlated to the abundance of *F. oxysporum* (*r* = 0.648, *P* = 0.001) as revealed by the Mantel test. PERMANOVA revealed that both the soil type and fumigation were significant drivers of the bacterial community composition (Table 1).

**Table 1:** Microbial community dissimilarity comparison among treatments using three non‐parametric statistical methods, including analysis of similarity(ANOSIM), multi‐response permutation procedure (MRPP) and non‐parametric multivariate analysis of variance (PERMANOVA)

Treatments	ANOSIM		MRPP		PERMANOVA	
	*R*	*P*	*A*	*P*	*F*	*P*
Rhizosphere vs. bulk	0.834	0.001	0.162	0.001	9.720	0.001
Fumigated vs. non‐fumigated	0.398	0.003	0.118	0.001	9.912	0.001

### Soil bacterial community structure

**Figure 2:**
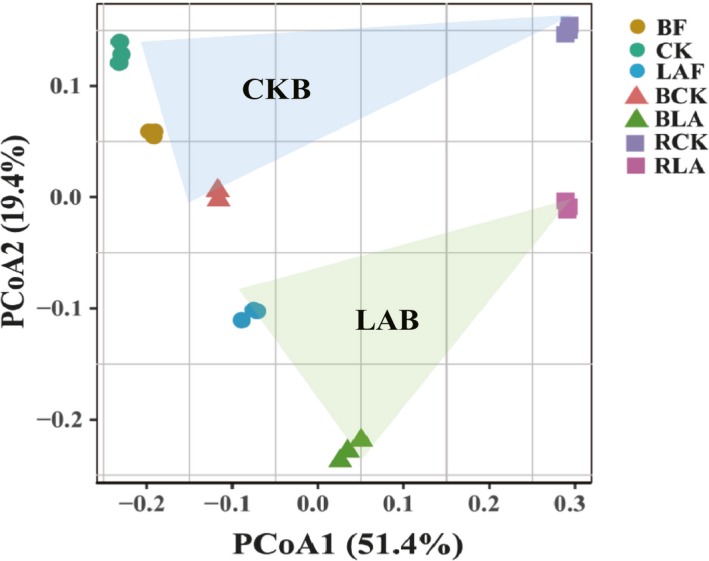
Principal coordination analysis (PCoA) plots of the bacterial community based on Bray–Curtis distance matrix. BF, CK and LAF represent the original soil samples before fumigation; soil samples were collected from non‐fumigation control and immediately after fumigation with lime and ammonium bicarbonate respectively. BCK and BLA represent bulk soil sample collected at the end of pot experiment of the LAB treatment and non‐fumigation control respectively. RCK and RLA represent rhizosphere soil sample collected at the end of pot experiment of the LAB treatment and CKB control respectively. CKB is treatment of biofertilizer applied to non‐fumigated control. LAB is treatment of biofertilizer applied to mixture of lime and ammonium bicarbonate fumigated soil.

Principal coordinate analysis (PCoA) based on the weighted UniFrac algorithm clearly revealed that the soil bacterial community varied across the soil types (rhizosphere vs. bulk soils and fumigated vs. non‐fumigated soils) (Fig. 2). The bulk soils treated with fumigants (LAF and BLA) separated from the non‐fumigated soils (BF, CK and BCK). The rhizosphere soils treated with fumigants (RLA) also separated distinctly from rhizosphere soils treated without non‐fumigants (RCK). The ANOSIM and MRPP analysis confirmed that soil type and fumigation were significant drivers in distinguishing the rhizosphere bacterial community structure (Table 1).

### Bacterial community diversity and phylogenetic relatedness

**Figure 3:**
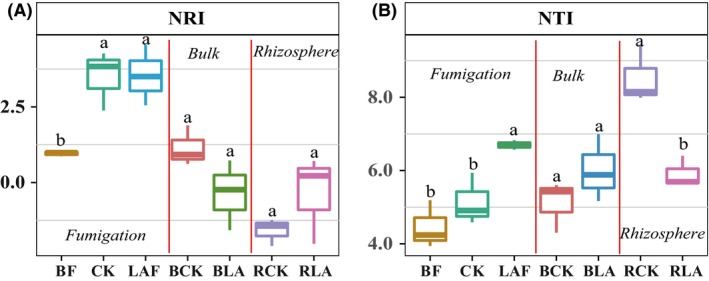
Boxplots of the phylogenetic relatedness based on nearest net relatedness (NRI) (A) and nearest taxon index (NTI) (B) of the bacterial community for different soil samples. BF, CK and LAF represent the original soil samples before fumigation; soil samples were collected from non‐fumigation control and immediately after fumigation with lime and ammonium bicarbonate respectively. BCK and BLA represent bulk soil sample collected at the end of pot experiment of the LAB treatment and non‐fumigation control respectively. RCK and RLA represent rhizosphere soil sample collected at the end of pot experiment of the LAB treatment and CKB control respectively. CKB is treatment of biofertilizer applied to non‐fumigated control. LAB is treatment of biofertilizer applied to mixture of lime and ammonium bicarbonate fumigated soil.

For soils sampled after fumigation, lime and ammonium bicarbonate fumigation significantly increased the nearest taxon index (NTI) compared to the non‐fumigated control (Fig. 3). At the end of pot experiment, the RLA soil showed a lower NTI compared to the RCK control while no significant difference was observed between bulk soil samples. Moreover, the nearest relatedness index (NRI) was much closer to zero for the rhizosphere soil samples compared to the non‐rhizosphere soil samples. Spearman correlation analysis revealed that the NRI was significantly and negatively correlated with the abundance of *F. oxysporum* (Fig. S3).

### Soil bacterial community composition

**Figure 4:**
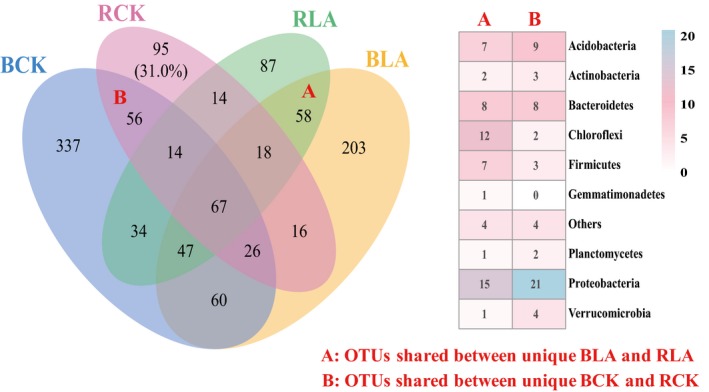
Venn diagram displaying the differences in the bacterial community composition for the bulk and rhizosphere soil samples collected at banana harvest for different treatments (left panel) (A). Heatmap showing the cumulative counts of OTUs affiliated to each phylum for unique OTUs between rhizosphere and bulk soil samples (B). ‘Others’ indicates phyla with an extremely low abundant. BCK and BLA represent bulk soil sample collected at the end of pot experiment of the LAB treatment and non‐fumigation control respectively. RCK and RLA represent rhizosphere soil sample collected at the end of pot experiment of the LAB treatment and CKB control respectively. CKB is treatment of biofertilizer applied to non‐fumigated control. LAB is treatment of biofertilizer applied to mixture of lime and ammonium bicarbonate fumigated soil.

Venn diagram showed that 295 OTUs and 441 OTUs were unique in bulk soils of the BLA and BCK treatments, respectively, while 226 OTUs and 193 OTUs were unique in rhizosphere soils of the RLA and RCK respectively (Fig. 4). Among these unique OTUs in rhizosphere soils, 58 in RLA and 56 in RCK were also observed as unique OTUs in bulk soil samples (BCK and BLA). Among those, more unique OTUs affiliated to Chloroflexi and Firmicutes were found in RLA compared to that in RCK treatment. Interestingly, unique potential biocontrol agents of OTU752, OTU4791, OTU277, OTU3815 and OTU3873 in BLA soil samples, classified as *Bacillus*,* Bacillus*,* Lysobacter*,* Lysobacter* and *Rhizobium*, respectively, were also detected in the RLA soil samples.

**Figure 5:**
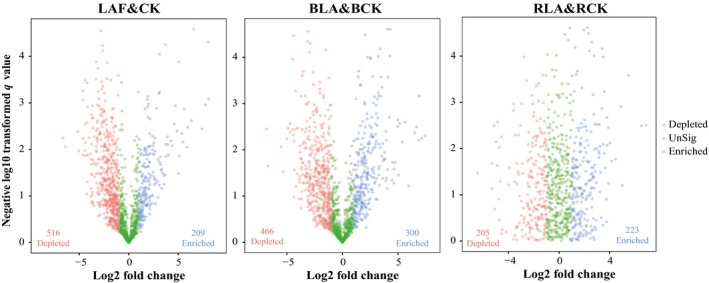
Volcanic plot displaying the fold change of OTUs in each soil sample based on data of log2 transformed from the relative abundance of each OTU. CK and LAF represent the original soil samples before fumigation; soil samples were collected from non‐fumigation control and immediately after fumigation with lime and ammonium bicarbonate respectively. BCK and BLA represent bulk soil sample collected at the end of pot experiment of the LAB treatment and non‐fumigation control respectively. RCK and RLA represent rhizosphere soil sample collected at the end of pot experiment of the LAB treatment and CKB control respectively. CKB is treatment of biofertilizer applied to non‐fumigated control. LAB is treatment of biofertilizer applied to mixture of lime and ammonium bicarbonate fumigated soil.

The relative abundances of bacterial phyla varied across soil samples collected during the fumigation and assembly stage (Fig. S2). The relative abundance of Actinobacteria, Chloroflexi, Firmicutes and Gemmatimonadetes was significantly negatively correlated with the abundance of *F. oxysporum* as revealed by the Spearman correlation analysis (Table S2). At the 97% identity level, the relative abundances of OTUs also varied with soil samples collected during the fumigation and assembly stage (Fig. 5). In detail, 725 OTUs in fumigated soils of LAF showed twofold changes compared to that in CK, among those, 209 OTUs were enriched after fumigation. After biofertilizer application and banana plantation, 766 OTUs in the bulk soil and 428 OTUs in the rhizosphere soil from LAB treatment still showed twofold changes compared to that in the CKB treatment. Among these OTUs with twofold changes, 300 OTUs in bulk soil and 223 OTUs in rhizosphere soil were enriched in LAB treatment.

**Figure 6:**
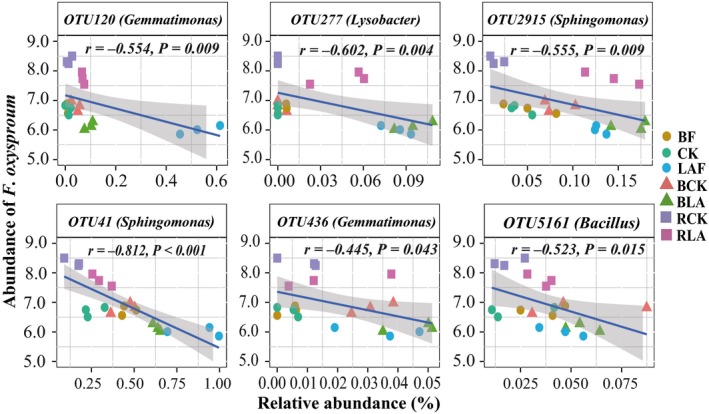
Spearman correlations between the abundance of *F. oxysporum* quantified by qPCR with the relative abundance of potential biocontrol agents. BF, CK and LAF represent the original soil samples before fumigation; soil samples were collected from non‐fumigation control and immediately after fumigation with lime and ammonium bicarbonate respectively. BCK and BLA represent bulk soil sample collected at the end of pot experiment of the LAB treatment and non‐fumigation control respectively. RCK and RLA represent rhizosphere soil sample collected at the end of pot experiment of the LAB treatment and CKB control respectively. CKB is treatment of biofertilizer applied to non‐fumigated control. LAB is treatment of biofertilizer applied to mixture of lime and ammonium bicarbonate fumigated soil.

On the other hand, 265 OTUs from the final OTU table showed significant negative correlations with the abundance of *F. oxysporum*. Taken fold changes and correlation analysis together, only 144 OTUs from negatively correlated OTUs were increased for the LAB treatment. However, only the following OTUs may be potentially associated with biocontrol. For example, OTU10 classified as *Rhizobium*, OTU120 and OTU436 classified as *Gemmatimonas*, OTU2915 and OTU41 classified as *Sphingomonas*, OTU130 classified as *Flavobacterium*, OTU14 classified as *Pseudomonas*, OTU277 classified as *Lysobacter*, and OTU561 classified as *Bacillus* were significantly increased after fumigation and/or further biofertilizer application in bulk soil and/or rhizosphere (Fig. S4). But only OTU120 (*Gemmatimonas*), OTU277 (*Lysobacter*), OTU2915 (*Sphingomonas*), OTU41 (*Pseudomonas*), OTU436 (*Gemmatimonas*) and OTU5161 (*Bacillus*) were significantly negatively correlated with the abundance of *F. oxysporum* (Fig. 6).

### Co‐occurrence patterns of potential biocontrol OTUs

Two distinct groups representing fumigated soil samples (LAF, BLA and RLA) or non‐fumigated soil samples (CK, BCK and RCK) were significantly different as confirmed by the ANOSIM (*r* = 0.398, *P *= 0.003) and MRPP (*A* = 0.118, *P *= 0.001) analysis (Table 1). The phylogenetic molecular ecological networks of 265 OTUs, which were negatively correlated to the abundance of *F. oxysporum*, were constructed using a random matrix theory‐based approach to explore the organization of these potential biocontrol agents in fumigated (LAB) or non‐fumigated (CKB) soil samples. After filtering OTUs that occurred in less than five samples for each treatment, 201 OTUs for fumigated samples (LAB) and 137 OTUs for non‐fumigated samples (CKB) were used to construct the networks.

**Figure 7:**
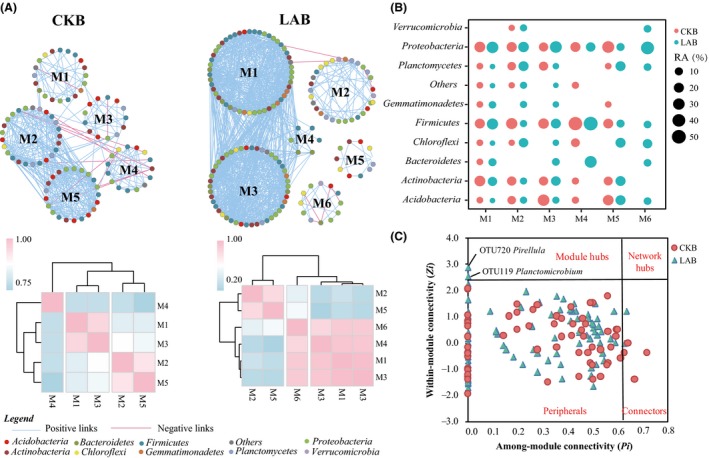
Co‐occurrence networks of up panel displaying the constructed network of soils sampled from biofertilizer applied to fumigated (LAB) and non‐fumigated soil (CKB) (A, left panel). Heatmap of down panel showing the correlations among the module eigengenes of each respective network (B). Blue colour indicates positive correlations, whereas pink signifies negative correlations. Modules containing larger than five nodes in the networks are labelled with corresponding letter followed by a number. Circular node colours indicate different bacterial phyla. Bubble graph showing the relative abundance of nodes in each module within each network at the phylum level (B, upper right panel). *Zi‐Pi* plot showing the distribution of OTUs based on their topological roles. Each symbol represents an OTU in CKB (red circle) or LAB (blue triangle) network (C, lower right panel). The threshold values of *Zi* and *Pi* for categorizing OTUs were 2.5 and 0.62 respectively.

The LAB network contained 170 nodes (OTUs), 932 edges and 12 modules, with an average connectivity (avgK) of 10.97 and clustering coefficient (avgCC) of 0.43 while the values in the CKB network were 107, 378, 10, 7.07 and 0.37 respectively (Table 2). More large modules (> 5 nodes) were identified in the LAB network than that in the CKB network. The module eigengene network analysis revealed a difference in the higher‐order organization between the two networks (Fig. 7A). The eigengenes of modules M2 and M5 clustered together as a meta‐module in the CKB network, while the eigengenes of module M1, M3 and M4 clustered together as a meta‐module in the LAB network. Notably, the node composition was substantially different between the two networks as the relative abundance of dominant phyla was obviously different among different modules (Fig. 7B). A higher ratio of OTUs affiliated to Bacteroidetes, Firmicutes, Gemmatimonadetes and Proteobacteria, and a lower ratio of OTUs affiliated to Acidobacteria and Planctomycetes within the meta‐modules was observed in the LAB versus CKB network.

**Table 2:** Major topological properties of phylogenetic molecular ecological networks of soil samples collected from biofertilizer applied to fumigated (LAB) and non‐fumigated (CKB) soil treatments

Network indexes	Network size	Total links	Average degree (avgK)	Average clustering coefficient (avgCC)	Average path distance (GD)	Modularity (No. of large modules)
CKB	107	378	7.07	0.37	3.74	0.69 (10)
LAB	170	932	10.97	0.43	3.67	0.77 (12)

CKB is treatment of biofertilizer applied to non‐fumigated control. LAB is treatment of biofertilizer applied to mixture of lime and ammonium bicarbonate fumigated soil.

Analysis using the threshold values of *Zi* and *Pi* showed that majority of nodes from both networks were categorized as peripherals that have only a few links and almost always link to the nodes within their own modules (Fig. 7C). Two nodes of OTU119 and OTU720, belonging to *Pirellula* and *Planctomicrobium* in Planctomycetes, were categorized as module hubs in the LAB network but were absent in the CKB network, suggesting that the hubs may be the key organisms to the communities. Interestingly, OTU119 was positively correlated with a common antifungal microorganism of OTU180 (*Bacillus*).

## Discussion

Plant microbiota could provide an accessory genome and reservoir of important functions to host plant and contribute to plant health (Sessitsch *et al*., 2018). Our previous study reported that biofertilizer applied to the soil fumigated with lime and ammonium bicarbonate effectively controlled the outbreak of banana Panama disease by manipulating the soil microbiome (Shen *et al*., 2018). However, plant rhizosphere is the key place for the invasion, colonization and reproduction of soil‐borne pathogens (Lennon and Jones, 2011). Considering bulk soil is a microbial seed bank of both pathogenic and beneficial microorganisms for the rhizosphere, we further hypothesized that the microbiome in the rhizosphere can also be steered due to the manipulated bulk soil microbiota. The study of how plant recruits beneficial microbes from bulk soil to assemble a disease‐suppressive microbiome in the rhizosphere during soil fumigation and biofertilization could help to explore the interactions between plant and rhizosphere microbiome. Thus, the changes of the bacterial community in the rhizosphere between treatments of biofertilizer applied to fumigated soil and non‐fumigated control were investigated during the second season under greenhouse condition.

### Manipulation of bulk soil microbiome induced a unique rhizosphere bacterial community

NRI in rhizosphere soils fluctuated near zero compared to non‐rhizosphere soils, indicating that the assembly of rhizosphere microbiome is driven by both deterministic and stochastic processes (Stegen *et al*., 2012, 2013; Ordonez *et al*., 2015). A significantly negative correlation of NRI with the abundance of *F. oxysporum* was found, suggesting that the assembled microbiome in rhizosphere may be involved in disease suppression. On the contrary, no significant correlation was observed between NTI and the abundance of *F. oxysporum*. This may be due to that NTI is more sensitive to terminal proximity topological branching, whereas NRI is based on mean pairwise distance of the terminal nodes across the whole tree and thus more sensitive to deeper topological branching (Kembel *et al*., 2011). However, a higher NTI was observed in RCK control, possibly due to a long‐term adaptation to mono‐cropping stress resulting in the dominance of closely related taxa (Kembel *et al*., 2011; Heděnec *et al*., 2018). Hence, soil microbiome manipulation may eliminate this mono‐cropping stress and re‐assemble the rhizosphere bacterial community to provide more niches for beneficial microbes.

The bacterial community composition was clearly separated between fumigated and non‐fumigated treatments in both bulk and rhizosphere soil. This roughly agrees with previous results that soil fumigation treated with rapeseed meal and broccoli residues (Wang *et al*., 2014) or ammonium bicarbonate and lime mixture (Liu *et al*., 2016) alter soil microbial community. Our results together with previous report (Shen *et al*., 2018) illustrated that soil fumigation could manipulate the dysbiosis of soil microbiome due to continuous cropping. Consistent with previous report that microbial community in rhizosphere could be shaped by microbiota in bulk soil (de Ridder‐Duine *et al*., 2005), our result also revealed that the variation of soil microbiome in bulk soil could still exhibit a strong effect on rhizosphere microbial community. Unlike previous study that direct application of antifungal biocontrol strain into soil displayed a minor effect on fungal communities (Antweiler *et al*., 2017), biofertilizer applied to fumigated soil displayed a strong influence on bacterial community composition both in bulk and rhizosphere soil even after banana re‐planted for couple of months. Thus, manipulation of the disturbed microbial community in bulk soil through lime and ammonium carbonate fumigation followed by biofertilizer application could assemble a healthy rhizosphere microbiome suppressive to banana Panama disease.

### Rhizosphere bacterial community suppressive to banana Panama disease was dominated by beneficial microbes

In the present study, lime and ammonium bicarbonate fumigation displayed a promising role in reducing *F. oxysporum* population in soil, which is in accordance with previous report that lime and ammonium bicarbonate fumigant application significantly decreased the abundance of *F. oxysporum* of cucumber by killing its mycelia (Liu *et al*., 2016). Moreover, biofertilizer application further suppressed the abundance of *F. oxysporum* further to a lower level even after banana was re‐planted, which is likely due to the disease‐suppressive capacity of the biofertilizer (Shen *et al*., 2015). Noteworthy, soil microbiome manipulation was effective in reducing the survival of *F. oxysporum* in bulk and rhizosphere soil even after banana replantation. Considering soils with lower disease incidence usually harboured a lower abundance of *F. oxysporum* (Ploetz, 2006), soil pathogen was still presented after fumigation and biofertilizer application indicating that the disease suppression may be also indirectly induced by the manipulated rhizosphere microbiome in this study.

Analysis of bacterial community composition revealed that several OTUs belonging to genera of *Gemmatimonas*,* Sphingomonas*,* Pseudomonas*,* Lysobacter* and *Bacillus* might be involved in disease suppression since they showed negative correlations to *F. oxysporum* and were enriched in bulk and/or rhizosphere soils after biofertilizer application in this study. This partly agrees with a common knowledge that disease suppression is most likely attributed to the complex soil microbial consortia (Mendes *et al*., 2011; Raaijmakers and Mazzola, 2016). Although the explicit disease‐suppressive function of *Gemmatimonas*, a widely existent genus in multiple terrestrial and aquatic habitats, is still unclear (Zhang *et al*., 2003), a higher abundance of *Gemmatimonas* was frequently detected in the healthy bulk and/or rhizosphere soils with low soil‐borne disease incidence (Yin *et al*., 2013; She *et al*., 2017). *Sphingomonas* is widely distributed in natural habitats, and recently, *Sphingomonas* was reported to be involved in disease management since it is responsible for *Fusarium* toxin degrading activity (He *et al*., 2017). *Pseudomonas* spp. has been utilized for banana Panama disease biocontrol (Saravanan *et al*., 2004; Kavino *et al*., 2010) as it could produce a wide spectrum of bioactive metabolites, colonize and multiply in the environment, and aggressively compete with other microorganisms (Weller, 1988; Lemanceau and Alabouvette, 1993). *Lysobacter* spp. may play an important role in the suppression of banana Panama disease since it could produce a wide range of extracellular enzymes and other metabolites with antagonistic activity against many soil‐borne pathogens (Expósito *et al*., 2015). As *Bacillus* spp. can secrete many antifungal compounds and form a stable and extensive biofilm that protect plants against the attack of soil‐borne pathogens (Bais *et al*., 2004; Yuan *et al*., 2012), it was likely to be involved in disease suppression in this study. Hence, these species may serve as key indicators of *Fusarium* wilt disease suppression in banana cropping systems and our findings suggest that maybe more attention should be paid about the disease suppressiveness of these candidate biocontrol consortia against soil‐borne disease.

### Beneficial microbes are well organized to suppress the possible pathogen invasion

Network analysis is a system‐level method to explore interactions within an ecosystem that cannot be directly observed through co‐occurrence analysis (Fath *et al*., 2007). Insight into the interactions within and between potential biocontrol species could improve our understanding of the changes in microbial consortia involved in disease suppression under banana monoculture. Overall, the topological properties of the constructed networks, including connectivity, average clustering coefficients, average degree distance and modularity, indicate that these networks are scale‐free, modular and ‘small world’ (Brown *et al*., 2004; Newman, 2006). Our comparative network analysis indicated that biofertilizer application and soil fumigation could influence microbial co‐occurrence patterns of potential functional species. A meta‐module is usually considered as a group of modules functionally interrelated (Langfelder and Horvath, 2007), a greater number of meta‐modules were identified in the network constructed from fumigated treatments, suggesting that a greater number of OTUs in the LAB network were functionally interrelated than that in the CKB network. This agrees with a previous report that a healthy community could be viewed as a better organized or a better operational community with more functionally interrelated members than a diseased community (Lu *et al*., 2013).

A majority of OTUs in the meta‐modules were not shared between LAB and CKB networks, indicating basal shifts in network architecture after soil microbiome manipulation. Moreover, Firmicutes and Gemmatimonadetes dominated meta‐modules were found in the LAB network, whereas Acidobacteria and Planctomycetes dominated meta‐modules were observed in the CKB network, illustrating a core bacterial community quasi‐functional shift (Hartman *et al*., 2018). Furthermore, more module hubs were present in disease‐suppressive network as revealed by the *Zi‐Pi* relationship of each individual OTU, which means that the community works more efficiently and the social goal is more likely to be achieved with more cooperation (Lu *et al*., 2013). Although module hubs in LAB network affiliated to Planctomycetes showed a negative correlation to pathogen abundance, there is still a lack in biological evidence to confirm this direct suppression. However, they could indirectly engine the common beneficial microorganisms (such as *Bacillus*) to directly suppress the pathogen abundance.

## Conclusion

The results from the present study demonstrated that the microbiome manipulation of bulk soil induced banana rhizosphere to assemble a bacterial community suppressive to banana Panama disease. The observed disease suppression may be due to the general suppression induced by fertilization and fumigation through (i) altering the soil community structure, (ii) increasing the phylogenetic relatedness, (iii) reducing the relative abundance of *F. oxysporum*, (iv) stimulating the indigenous beneficial microbial consortia (e.g. *Gemmatimonas*,* Sphingomonas*,* Pseudomonas*,* Lysobacter* and *Bacillus*) and (v) interrelating and better organizing the potential species involved in disease suppression.

## Experimental procedures

### Pot experiment description

Two‐season pot experiment was conducted in the greenhouse of WanZhong Agricultural Company, in Jianfeng, Hainan province, China, during June–November 2012 and January–May 2013 (Table 3, Fig. S1). The fumigation and biofertilizer application were performed for each season. Two treatments were established within each season including (i) biofertilizer applied to soil fumigated by lime and ammonium bicarbonate (LAB) and (ii) biofertilizer applied to non‐fumigated soil (CKB). The fumigant and biofertilizer used for each treatment are provided in Table 3. Topsoils used for the pot experiment were collected from the banana mono‐cropped field (18°38′N, 108°47′E) in WanZhong Agricultural Company. The field was under continuous banana cultivation for more than 15 years and showed more than 50% of Panama disease incidence with approximately 10^5^ CFU of *F. oxysporum* per gram soil. The soil has a pH value of 6.15, organic matter content of 6.25 g kg^−1^, a total N content of 0.87 g kg^−1^ and available P, K contents of 76, 126 mg kg^−1^ respectively. The soil is classified as loam sandy which developed from dry red soil. The detail information regarding experimental settings, fumigation processing and fertilization regimes was provided in our previous study (Shen *et al*., 2018).

**Table 3:** Detail information of the fumigant and fertilizers used for each treatment in the experiment

Treatment	Soil sample	Description	Amounts of fumigant (g/6 kg soil)		Amounts of fertilizer (g/6 kg soil)	
			Lime	Ammonium bicarbonate	Base fertilizer	After manuring
CKB	BF	Original soil			120	60
	CK	Non‐fumigated control soil				
	BCK	Bulk soil collected from treatments of biofertilizer applied to non‐fumigated soil				
	RCK	Rhizosphere soil collected from treatments of biofertilizer applied to non‐fumigated soil				
LAB	LAF	Mixture of lime and ammonium bicarbonate fumigated soil	20	10	120	60
	BLA	Bulk soil collected from treatments of biofertilizer applied to fumigated soil				
	RLA	Rhizosphere soil collected from treatments of biofertilizer applied to fumigated soil				

CKB is treatment of biofertilizer application to non‐fumigated control. LAB is treatment of biofertilizer application to mixture of lime and ammonium bicarbonate fumigated soil. BF, CK and LAF represent the original soil sample before fumigation; soil sample immediately collected after fumigation with lime and ammonium bicarbonate, and non‐fumigation control respectively. BCK and BLA represent bulk soil sample collected at the end of pot experiment of the LAB treatment and non‐fumigation control respectively. RCK and RLA represent rhizosphere soil sample collected at the end of pot experiment of the LAB treatment and non‐fumigation control respectively.

### Soil sampling and DNA extraction

Soil sampling was performed during the second season of the greenhouse experiment between January and May 2013. One kilogram of the original soil before fumigation (BF) was sampled at the beginning of greenhouse experiment. Bulk soil was sampled and processed according to our previous study (Shen *et al*., 2018). For rhizosphere soil sample collection, five banana plants without obvious infection symptoms from each replicate were randomly selected. All roots from each replicate were pooled together, shaken slightly by hands to remove the loosely adhered soil and minced approximately into 5 cm long. After that, about 200 g of roots was added to a 1 l Erlenmeyer flask containing 250 ml of sterilized distilled water. Finally, the soil suspension was centrifuged at 4000 × *g* for 5 min after shaking for 30 min at 170 rpm under ambient temperature. The precipitated soil was collected as rhizosphere samples related to biofertilizer applied to fumigated soil (RLA) and applied to non‐fumigated soil (RCK). In total, 21 soil samples from seven treatments were sampled, including original soil before fumigation (BF), lime and ammonium bicarbonate fumigated soil (LAF) and non‐fumigated control (CK) immediately collected after fumigation, bulk soil for biofertilizer applied to fumigated soil (BLA) or non‐fumigated control (BCK) and rhizosphere soil for biofertilizer applied to fumigated soil (RLA) or non‐fumigated control soil (RCK) sampled after banana planted for 4 months. Genomic DNA of each sample was extracted by the PowerSoil DNA Isolation Kit according to the manual (MoBio Laboratories, Carlsbad, CA, USA) and stored at −70°C for further PCR amplification.

### Quantification of *F. oxysporum*

The abundance of *F*. *oxysporum* in soil was determined using the primers *FocSc*‐1/*FocSc*‐2 on the 7500 Real‐Time PCR System (Applied Biosystems, Foster City, CA, USA) as described by Huang *et al*. (2015). Standard curves were constructed using 10‐fold serial dilutions of a plasmid containing a copy of the ITS fragment from *Foc*4. Quantitative PCR amplifications were performed in a 20‐μl mixture for each reaction containing 2 μl template DNA, 10 μl SYBR^®^Premix Ex Taq™ (TaKaRa, Shiga, Japan), 0.4 μl of each primer, 0.4 μl ROX Reference Dye II and nuclease‐free water. Thermal cycling conditions for each sample with three technical replicates were run according to a standard procedure, and the results were expressed as log copy numbers g^−1^ dry soil.

### Illumina library construction and sequencing

Bacterial sequencing libraries were constructed according to the procedure described by Caporaso *et al*. (2011). Briefly, the V4 region of bacterial 16S rRNA genes was amplified by the primers 515F/806R using the ThermoScientific^®^Phusion High‐Fidelity PCR Master Mix (New England Biolabs, Ipswich, MA, USA). All amplifications were conducted in a 30 μl mixture including 15 μl of 2 × Master mix, 0.5 μM final concentration of forward and reverse primers, 10 ng of template DNA and nuclease‐free water. The PCR products were purified using PCR Purification Kit (QIAGEN, Hilden, Germany) and quantified using Qubit^®^ 2.0 Fluorometer (Invitrogen, Carlsbad, CA, USA). The purified amplicons were employed for library construction with the NEB Next^®^ Ultra™ DNA Library Prep Kit (New England Biolabs). The final library was quality‐assessed with Agilent 2100 Bioanalyzer Instruments (Agilent Technologies, Palo Alto, CA, USA) and quantified using KAPA Library Quantification Kits (Kapa Biosystems, Wilmington, MA, USA). All constructed libraries were sequenced by Illumina HiSeq 2000 at Novogene Bioinformatics Institute (Beijing, China).

### Sequence data processing

Raw sequences were assigned to individual samples according to the unique barcode and the adaptor and primer sequences were trimmed in QIIME (Caporaso *et al*., 2010). After removing low‐quality sequences, single‐stranded sequences of forward and reverse direction were merged. The merged sequences were processed using the UPARSE pipeline to generate operational taxonomic units (OTUs) (Edgar, 2013). A representative sequence for each OTU was selected and classified using the RDP classifier against the RDP Bacterial 16S database (Wang *et al*., 2007). OTUs affiliated to archaea were removed from the OTU table. Only OTUs with abundances higher than 0.01% in at least one sample were kept for the downstream analysis. The relative abundance of each OTU was calculated as the number of sequences affiliated to the target OTU divided by the total number of sequences in individual samples. All raw sequences were deposited in NCBI under the accession number PRJNA493948 and PRJNA393952.

### Statistical analyses

Data were square‐root or log‐transformed to meet the criteria of normal distribution when necessary. Principal coordinate analysis (PCoA) based on weighted UniFrac metric was performed to evaluate the differences in bacterial communities using the ‘vegan’ package in the R platform. To compare the bacterial community composition among treatments, a Venn diagram was generated based on the shared OTU table from the subsample after removing singletons. Fold change (log2 transformed) was also calculated to evaluate the differences of the bacterial community composition across different treatments. Co‐occurrence network of OTUs, which were negatively correlated to the abundance of *F. oxysporum*, was determined by modelling the microbial community using molecular ecological network analysis (http://ieg2.ou.edu/MENA) (Zhou *et al*., 2011) for soil samples collected after fumigation and biofertilization from the LAB and CKB treatments. The microbial network was constructed using the random matrix theory at 0.94 similarity threshold and visualized using Cytoscape 2.8.3 software (http://cytoscape.org/). Nearest relatedness index (NRI) and nearest taxon index (NTI), the two most widely used indices, were selected to evaluate the assembly process of microbial community in this study. Microbial community phylogenetic structure was examined with NRI and NTI using the null model ‘independent swap’ (Gotelli and Entsminger, 2003) with 999 randomization runs with 999 iterations in R using the ‘Picante’ package (Kembel *et al*., 2010).

To determine significant differences, multiplicity analysis of variance or two‐tailed, unpaired t‐tests and one‐way ANOVA were performed. Spearman correlations among diversity indices, individual microbial groups and the abundance of *F. oxysporum* were tested in R. Mantel tests were used to identify correlations between the bacterial community composition and the abundance of *F. oxysporum* using the ‘vegan’ package in R. Analysis of similarities among treatments (ANOSIM), multi‐response permutation procedure (MRPP) and non‐parametric multivariate analysis of variance (PERMANOVA) were performed to test the significant differences using the ‘vegan’ package.

## Conflict of interests

The authors declare that there is no conflict of interests.

## Ethical approval

This article does not contain any studies with animals performed by any of the authors.

## Supporting information


**Fig. S1.** Sketch map for each season of the two seasonal greenhouse experiments.

**Fig. S2.** Heatmap displaying the abundance of dominated bacterial phyla in each soil sample.

**Fig. S3.** Spearman correlations between the abundance of *F. oxysproum* quantified by qPCR with the phylogenetic relatedness of based on nearest net relatedness (NRI) (a) and nearest taxon index (NTI) (b) in bacterial community.

**Fig. S4.** Boxplot of the relative abundance of OTU10 (*Rhizobium*), OTU120 (*Gemmatimonas*), OTU130 (*Flavobacterium*), OTU14 (*Pseudomonas*), OTU277 (*Lysobacter*), and OTU2915 (*Sphingomonas*) for each soil sample.

**Table S1.** Number of sequences and OTUs from rawdata and processed final good quality sequences that were used to further analysis after basic quality control for different treatments.

**Table S2.** Spearman correlation coefficients and significant *p* value between the relative abundance of *F. oxysproum* with abundant bacterial and fungal phyla.

## References

[mbt213391-bib-0001] Antweiler, K. , Schreiter, S. , Keilwagen, J. , Baldrian, P. , Kropf, S. , Smalla, K. , *et al* (2017) Statistical test for tolerability of effects of an antifungal biocontrol strain on fungal communities in three arable soils. Microb Biotechnol 10: 434–449. 28111906 10.1111/1751-7915.12595 PMC5328832

[mbt213391-bib-0002] Bais, H.P. , Fall, R. , and Vivanco, J.M. (2004) Biocontrol of *Bacillus subtilis* against infection of *Arabidopsis* roots by *Pseudomonas syrin gae* is facilitated by biofilm formation and surfactin production. Plant Physiol 134: 307–319. 14684838 10.1104/pp.103.028712 PMC316310

[mbt213391-bib-0003] Bais, H.P. , Weir, T.L. , Perry, L.G. , Gilroy, S. , and Vivanco, J.M. (2006) The role of root exudates in rhizosphere interactions with plants and other organisms. Annu Rev Plant Biol 57: 233–266. 16669762 10.1146/annurev.arplant.57.032905.105159

[mbt213391-bib-0004] Berg, G. , and Smalla, K. (2009) Plant species and soil type cooperatively shape the structure and function of microbial communities in the rhizosphere. FEMS Microbiol Ecol 68: 515–527. 10.1111/j.1574-6941.2009.00654.x 19243436

[mbt213391-bib-0005] Brown, K.S. , Hill, C.C. , Calero, G.A. , Myers, C.R. , Lee, K.H. , Sethna, J.P. , and Cerione, R.A. (2004) The statistical mechanics of complex signaling networks: nerve growth factor signaling. Phys Biol 1: 184. 16204838 10.1088/1478-3967/1/3/006

[mbt213391-bib-0006] Butler, D. (2013) Fungus threatens top banana. Nature 504: 195–196. 24336262 10.1038/504195a

[mbt213391-bib-0007] Caporaso, J.G. , Kuczynski, J. , Stombaugh, J. , Bittinger, K. , Bushman, F.D. , Costello, E.K. , *et al* (2010) QIIME allows analysis of high‐throughput community sequencing data. Nat Methods 7: 335–336. 20383131 10.1038/nmeth.f.303 PMC3156573

[mbt213391-bib-0008] Caporaso, J.G. , Lauber, C.L. , Walters, W.A. , Berg‐Lyons, D. , Lozupone, C.A. , Turnbaugh, P.J. , *et al* (2011) Global patterns of 16S rRNA diversity at a depth of millions of sequences per sample. Proc Natl Acad Sci USA 108: 4516–4522. 20534432 10.1073/pnas.1000080107 PMC3063599

[mbt213391-bib-0009] Chaparro, J.M. , Sheflin, A.M. , Manter, D.K. , and Vivanco, J.M. (2012) Manipulating the soil microbiome to increase soil health and plant fertility. Biol Fert Soils 48: 489–499.

[mbt213391-bib-0010] Chase, J.M. (2007) Drought mediates the importance of stochastic community assembly. Proc Natl Acad Sci USA 104: 17430–17434. 17942690 10.1073/pnas.0704350104 PMC2077273

[mbt213391-bib-0011] Cook, R.J. , Thomashow, L.S. , Weller, D.M. , Fujimoto, D. , Mazzola, M. , Bangera, G. , and Kim, D.S. (1995) Molecular mechanisms of defense by rhizobacteria against root disease. Proc Natl Acad Sci USA 92: 4197–4201. 11607544 10.1073/pnas.92.10.4197 PMC41910

[mbt213391-bib-0012] Deltour, P. , França, S.C. , Pereira, O.L. , Cardoso, I. , de Neve, S. , Debode, J. , and Höfte, M. (2017) Disease suppressiveness to *Fusarium* wilt of banana in an agroforestry system: influence of soil characteristics and plant community. Agric Ecosyst Environ 239: 173–181.

[mbt213391-bib-0013] Dini‐Andreote, F. , Stegen, J.C. , van Elsas, J.D. , and Falcão, S.J. (2015) Disentangling mechanisms that mediate the balance between stochastic and deterministic processes in microbial succession. Proc Natl Acad Sci USA 112: E1326–E1332. 25733885 10.1073/pnas.1414261112 PMC4371938

[mbt213391-bib-0014] Edgar, R.C. (2013) UPARSE: highly accurate OTU sequences from microbial amplicon reads. Nat Methods 10: 996–998. 23955772 10.1038/nmeth.2604

[mbt213391-bib-0015] Evans, S. , and Wallenstein, M. (2014) Climate change alters ecological strategies of soil bacteria. Ecol Lett 17: 155–164. 24261594 10.1111/ele.12206

[mbt213391-bib-0016] Expósito, G.R. , Postma, J. , Raaijmakers, J.M. , and Bruijn, I.D. (2015) Diversity and activity of *Lysobacter* species from disease suppressive soils. Front Microbiol 6: 1243. 26635735 10.3389/fmicb.2015.01243 PMC4644931

[mbt213391-bib-0017] Food and Agriculture Organization of the United Nations (FAOSTAT ) (2017) Data of crop production. URL http://www.fao.org/faostat/en/#data/QC.

[mbt213391-bib-0018] Fath, B.D. , Scharler, U.M. , Ulanowicz, R.E. , and Hannon, B. (2007) Ecological network analysis: network construction. Ecol Model 208: 49–55.

[mbt213391-bib-0019] Gotelli, N.J. , and Entsminger, G.L. (2003) Swap algorithms in null model analysis. Ecology 84: 532–535. 10.1007/s004420100717 28547607

[mbt213391-bib-0020] Haichar, F.E.Z. , Marol, C. , Berge, O. , Rangel‐Castro, J.I. , Prosser, J.I. , Balesdent, J. , *et al* (2008) Plant host habitat and root exudates shape soil bacterial community structure. ISME J 2: 1221–1230. 18754043 10.1038/ismej.2008.80

[mbt213391-bib-0021] Hartman, K. , van der Heijden, M.G. , Wittwer, R.A. , Banerjee, S. , Walser, J.C. , and Schlaeppi, K. (2018) Cropping practices manipulate abundance patterns of root and soil microbiome members paving the way to smart farming. Microbiome 6: 14. 29338764 10.1186/s40168-017-0389-9 PMC5771023

[mbt213391-bib-0022] He, W.J. , Zhang, L. , Yi, S.Y. , Tang, X.‐L. , Yuan, Q.‐S. , Guo, M.‐W. , *et al* (2017) An aldo‐keto reductase is responsible for *Fusarium* toxin‐degrading activity in a soil *Sphingomonas* strain. Sci Rep 7: 9549. 28842569 10.1038/s41598-017-08799-w PMC5573404

[mbt213391-bib-0023] Heděnec, P. , Rui, J. , Lin, Q. , Lin, Q. , Yao, M. , Li, J. , *et al* (2018) Functional and phylogenetic response of soil prokaryotic community under an artificial moisture gradient. Appl Soil Ecol 124: 372–378.

[mbt213391-bib-0024] Huang, X.Q. , Wen, T. , Zhang, J.B. , Meng, L. , Zhu, T.B. , and Liu, L.L. (2015) Control of soil‐borne pathogen *Fusarium oxysporum* by biological soil disinfestation with incorporation of various organic matters. Eur J Plant Pathol 143: 223–235.

[mbt213391-bib-0025] Kavino, M. , Harish, S. , Kumar, N. , Saravanakumar, D. , and Samiyappan, R. (2010) Effect of chitinolytic PGPR on growth, yield and physiological attributes of banana (*Musa* spp.) under field conditions. Appl Soil Ecol 45: 71–77.

[mbt213391-bib-0026] Kembel, S.W. , Cowan, P.D. , Helmus, M.R. , Cornwell, W.K. , Morlon, H. , Ackerly, D.D. , *et al* (2010) Picante: R tools for integrating phylogenies and ecology. Bioinformatics 26: 1463–1464. 20395285 10.1093/bioinformatics/btq166

[mbt213391-bib-0027] Kembel, S.W. , Eisen, J.A. , Pollard, K.S. , and Green, J.L. (2011) The phylogenetic diversity of metagenomes. PLoS ONE 6: e23214. 21912589 10.1371/journal.pone.0023214 PMC3166145

[mbt213391-bib-0028] Langfelder, P. , and Horvath, S. (2007) Eigengene networks for studying the relationships between co‐expression modules. BMC Syst Biol 1: 54. 18031580 10.1186/1752-0509-1-54 PMC2267703

[mbt213391-bib-0029] Lemanceau, P. , and Alabouvette, C. (1993) Suppression of Fusarium wilts by fluorescent pseudomonas: mechanisms and applications. Biocontrol Sci Technol 3: 219–234.

[mbt213391-bib-0030] Lennon, J.T. , and Jones, S.E. (2011) Microbial seed banks: the ecological and evolutionary implications of dormancy. Nat Rev Microbiol 9: 119–130. 21233850 10.1038/nrmicro2504

[mbt213391-bib-0031] Liu, L. , Sun, C. , Liu, X. , He, X. , Liu, M. , Wu, H. , Tang, C. , Jin, C. , and Zhang, Y. (2016) Effect of calcium cyanamide, ammonium bicarbonate and lime mixture, and ammonia water on survival of *Ralstonia solanacearum* and microbial community. Scie Rep 6: 19037. 10.1038/srep19037 PMC4704052 26738601

[mbt213391-bib-0032] Lu, L. , Yin, S. , Liu, X. , Zhang, W. , Gu, T. , Shen, Q. , and Qiu, H. (2013) Fungal networks in yield‐invigorating and‐debilitating soils induced by prolonged potato monoculture. Soil Biol Biochem 65: 186–194.

[mbt213391-bib-0033] Lundberg, D.S. , Lebeis, S.L. , Paredes, S.H. , Yourstone, S. , Gehring, J. , Malfatti, S. , *et al* (2012) Defining the core *Arabidopsis thaliana* root microbiome. Nature 488: 86–90. 22859206 10.1038/nature11237 PMC4074413

[mbt213391-bib-0034] Mendes, R. , Kruijt, M. , de Bruijn, I. , Dekkers, E. , van der Voort, M. , Schneider, J.H. , *et al* (2011) Deciphering the rhizosphere microbiome for disease‐suppressive bacteria. Science 332: 1097–1100. 21551032 10.1126/science.1203980

[mbt213391-bib-0035] Nemergut, D.R. , Schmidt, S.K. , Fukami, T. , O'Neill, S.P. , Bilinski, T.M. , Stanish, L.F. , *et al* (2013) Patterns and processes of microbial community assembly. Microbiol Mol Biol Rev 77: 342–356. 24006468 10.1128/MMBR.00051-12 PMC3811611

[mbt213391-bib-0036] Newman, M.E. (2006) Modularity and community structure in networks. Proc Natl Acad Sci USA 103: 8577–8582. 16723398 10.1073/pnas.0601602103 PMC1482622

[mbt213391-bib-0037] Ordonez, N. , Seidl, M.F. , Waalwijk, C. , Drenth, A. , Kilian, A. , Thomma, B.P. , *et al* (2015) Worse comes to worst: bananas and Panama disease‐when plant and pathogen clones meet. PLoS Pathog 11: e1005197. 26584184 10.1371/journal.ppat.1005197 PMC4652896

[mbt213391-bib-0038] Philippot, L. , Raaijmakers, J.M. , Lemanceau, P. , and van der Putten, W.H. (2013) Going back to the roots: the microbial ecology of the rhizosphere. Nat Rev Microbiol 11: 789–799. 24056930 10.1038/nrmicro3109

[mbt213391-bib-0039] Ploetz, R.C. (2006) Fusarium wilt of banana is caused by several pathogens referred to as *Fusarium oxysporum* f. sp. *cubense* . Phytopathology 96: 653–656. 18943184 10.1094/PHYTO-96-0653

[mbt213391-bib-0040] Ploetz, R.C. (2015) Management of Fusarium wilt of banana: a review with special reference to tropical race 4. Crop Prot 73: 7–15.

[mbt213391-bib-0041] Raaijmakers, J.M. , and Mazzola, M. (2016) Soil immune responses. Science 352: 1392–1393. 27313024 10.1126/science.aaf3252

[mbt213391-bib-0042] Raaijmakers, J.M. , Paulitz, T.C. , Steinberg, C. , Alabouvette, C. , and Moënne‐Loccoz, Y. (2009) The rhizosphere: a playground and battlefield for soilborne pathogens and beneficial microorganisms. Plant Soil 321: 341–361.

[mbt213391-bib-0043] de Ridder‐Duine, A. , Kowalchuk, G.A. , Gunnewiek, P.J.A.K. , Smant, W. , van Veen, J.A. , and de Boer, W. (2005) Rhizosphere bacterial community composition in natural stands of *Carex arenaria* (sand sedge) is determined by bulk soil community composition. Soil Biol Biochem 37: 349–357.

[mbt213391-bib-0044] Saravanan, T. , Muthusamy, M. , and Marimuthu, T. (2004) Effect of *Pseudomonas fluorescens* on *Fusarium* wilt pathogen in banana rhizosphere. Int J Biol Sci 4: 192–198.

[mbt213391-bib-0045] Sessitsch, A. , Brader, G. , Pfaffenbichler, N. , Gusenbauer, D. , and Mitter, B. (2018) The contribution of plant microbiota to economy growth. Microb Biotechnol 11: 801–805. 29926519 10.1111/1751-7915.13290 PMC6116737

[mbt213391-bib-0046] She, S. , Niu, J. , Zhang, C. , Xiao, Y. , Chen, W. , Dai, L. , *et al* (2017) Significant relationship between soil bacterial community structure and incidence of bacterial wilt disease under continuous cropping system. Arch Microbiol 199: 267–275. 27699437 10.1007/s00203-016-1301-x

[mbt213391-bib-0047] Shen, Z. , Ruan, Y. , Chao, X. , Zhang, J. , Li, R. , and Shen, Q. (2015) Rhizosphere microbial community manipulated by 2 years of consecutive biofertilizer application associated with banana *Fusarium* wilt disease suppression. Biol Fert Soils 51: 553–562.

[mbt213391-bib-0048] Shen, Z. , Xue, C. , Taylor, P.W.J. , Ou, Y. , Wang, B. , Zhao, Y. , *et al* (2018) Soil pre‐fumigation could effectively improve the disease suppressiveness of biofertilizer to banana *Fusarium* wilt disease by reshaping the soil microbiome. Biol Fert Soils 54: 793–806.

[mbt213391-bib-0049] Stegen, J.C. , Lin, X. , Konopka, A.E. , and Fredrickson, J.K. (2012) Stochastic and deterministic assembly processes in subsurface microbial communities. ISME J 6: 1653–1664. 22456445 10.1038/ismej.2012.22 PMC3498916

[mbt213391-bib-0050] Stegen, J.C. , Lin, X. , Fredrickson, J.K. , Chen, X. , Kennedy, D.W. , Murray, C.J. , *et al* (2013) Quantifying community assembly processes and identifying features that impose them. ISME J 7: 2069–2079. 23739053 10.1038/ismej.2013.93 PMC3806266

[mbt213391-bib-0051] Tilman, D. (2004) Niche tradeoffs, neutrality, and community structure: a stochastic theory of resource competition, invasion, and community assembly. Proc Natl Acad Sci USA 101: 10854–10861. 15243158 10.1073/pnas.0403458101 PMC503710

[mbt213391-bib-0052] Vellend, M. (2010) Conceptual synthesis in community ecology. Q Rev Biol 85: 183–206. 20565040 10.1086/652373

[mbt213391-bib-0053] Wang, Q. , Garrity, G.M. , Tiedje, J.M. , and Cole, J.R. (2007) Naive Bayesian classifier for rapid assignment of rRNA sequences into the new bacterial taxonomy. Appl Environ Microbiol 73: 5261–5267. 17586664 10.1128/AEM.00062-07 PMC1950982

[mbt213391-bib-0054] Wang, Q. , Ma, Y. , Yang, H. , and Chang, Z. (2014) Effect of biofumigation and chemical fumigation on soil microbial community structure and control of pepper *Phytophthora blight* . World J Microbiol Biotechnol 30: 507–518. 23990067 10.1007/s11274-013-1462-6

[mbt213391-bib-0055] Webb, C.O. (2000) Exploring the phylogenetic structure of ecological communities: an example for rain forest trees. Am Nat 156: 145–155. 10856198 10.1086/303378

[mbt213391-bib-0056] Webb, C.O. , Ackerly, D.D. , McPeek, M.A. , and Donoghue, M.J. (2003) Phylogenies and community ecology. Ann Rev Ecology Syst 33: 475–505.

[mbt213391-bib-0057] Weller, D.M. (1988) Biological control of soilborne plant pathogens in the rhizosphere with bacteria. Ann Rev Phytopathol 26: 379–407.

[mbt213391-bib-0058] Yin, C. , Hulbert, S.H. , Schroeder, K.L. , Mavrodi, O. , Mavrodi, D. , Dhingra, A. , *et al* (2013) Role of bacterial communities in the natural suppression of *Rhizoctonia solani* bare patch of wheat (*Triticum aestivum* L.). Appl Environ Microbiol 79: 7428–7438. 24056471 10.1128/AEM.01610-13 PMC3837727

[mbt213391-bib-0059] Yuan, J. , Li, B. , Zhang, N. , Raza, W. , Shen, Q. , and Huang, Q. (2012) Production of bacillomycin‐and macrolactin‐type antibiotics by *Bacillus amyloliquefaciens* NJN‐6 for suppressing soilborne plant pathogens. J Agr Food Chem 60: 2976–2981. 22385216 10.1021/jf204868z

[mbt213391-bib-0060] Zhang, H. , Sekiguchi, Y. , Hanada, S. , Hugenholtz, P. , Kim, H. , Kamagata, Y. , and Nakamura, K. (2003) *Gemmatimonas aurantiaca* gen. nov., sp. nov., a Gram‐negative, aerobic, polyphosphate‐accumulating micro‐organism, the first cultured representative of the new bacterial phylum *Gemmatimonadetes* phyl. nov. Int J Syst Evol Microbiol 53: 1155–1163. 12892144 10.1099/ijs.0.02520-0

[mbt213391-bib-0061] Zhou, J. , Deng, Y. , Luo, F. , He, Z. , and Yang, Y. (2011) Phylogenetic molecular ecological network of soil microbial communities in response to elevated CO_2_ . mBio 2: e00122‐00111. 21791581 10.1128/mBio.00122-11 PMC3143843

