# Supplementary figures and images for: Lime and ammonium carbonate fumigation coupled with bio‐organic fertilizer application steered banana rhizosphere to assemble a unique microbiome against Panama disease
Source: Microb Biotechnol. 2019 Mar 5;12(3):515–27. doi: 10.1111/1751-7915.13391 (PMC6465235; doi:10.1111/1751-7915.13391)

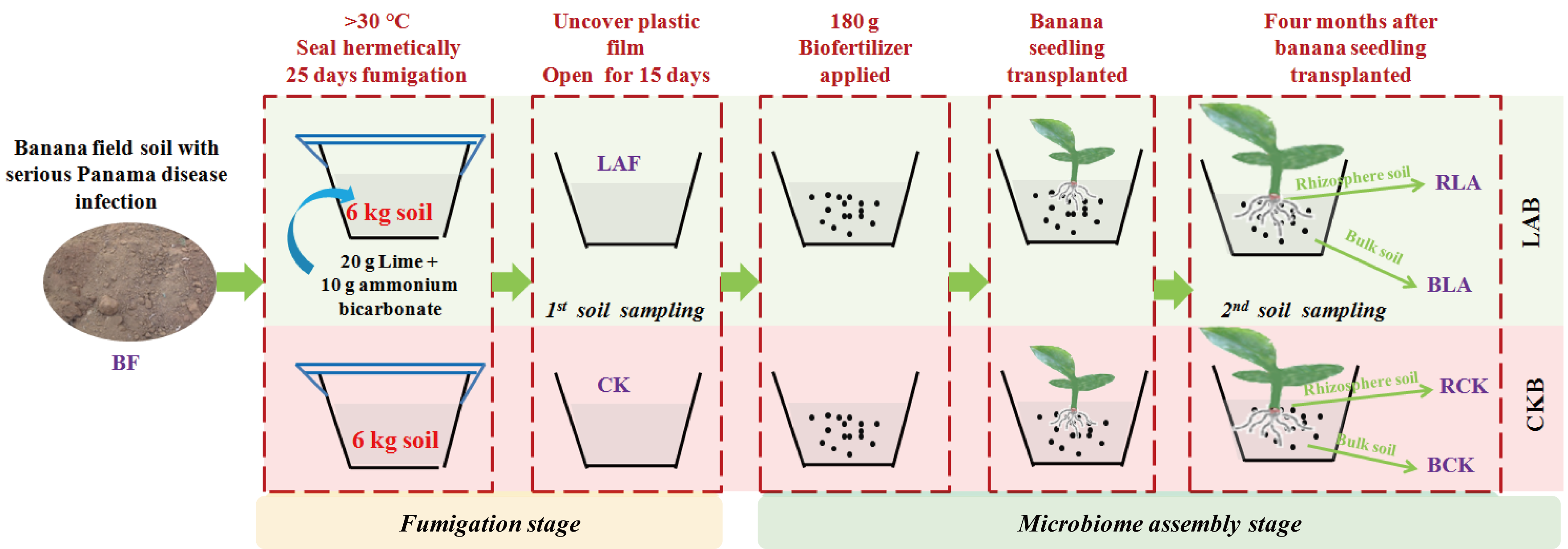

Supplement: Supplementary file 1 — Fig. S1. Sketch map for each season of the two seasonal greenhouse experiments. [file MBT2-12-515-s001.tif]

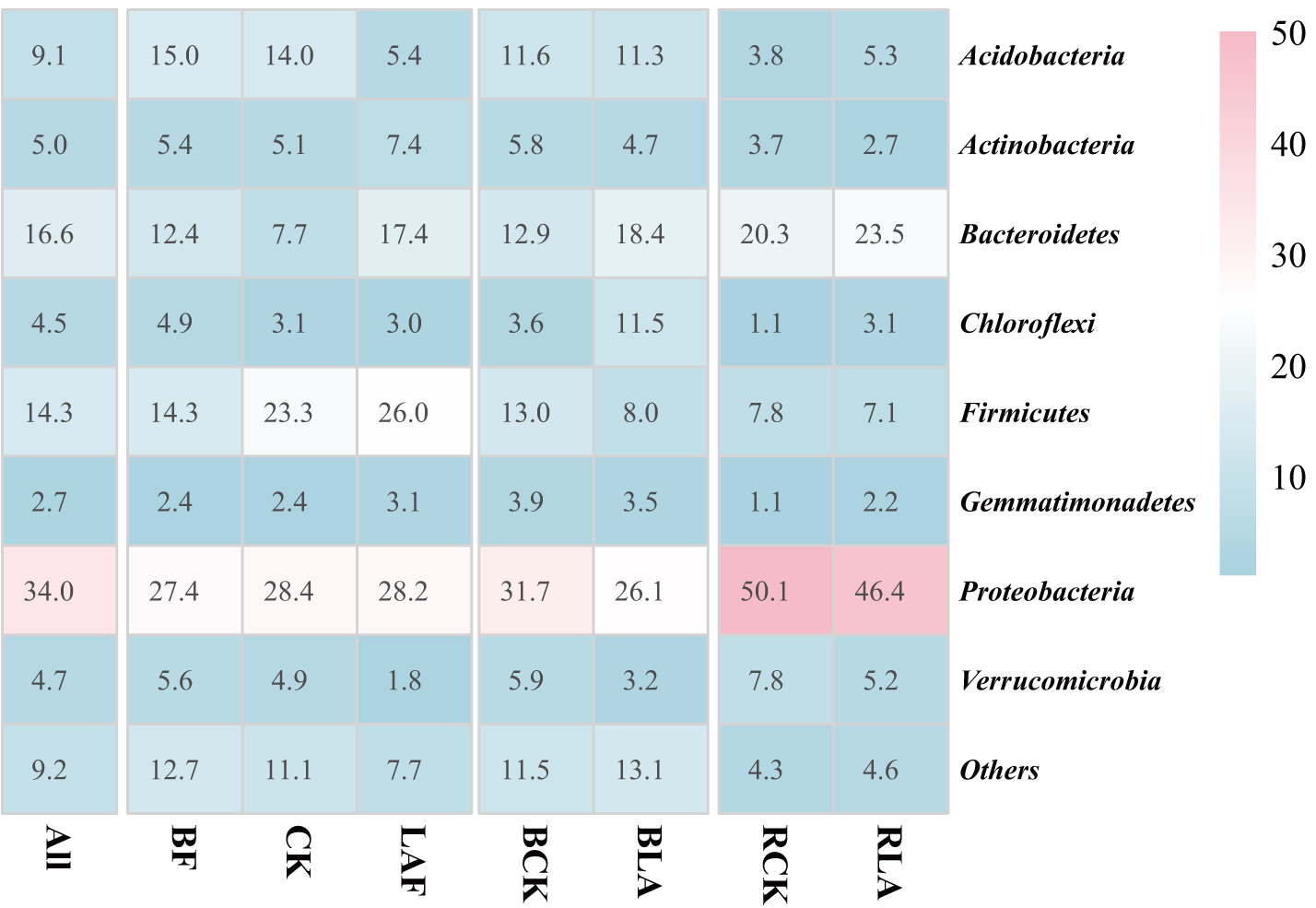

Supplement: Supplementary file 2 — Fig. S2. Heatmap displaying the abundance of dominated bacterial phyla in each soil sample. [file MBT2-12-515-s002.tif]

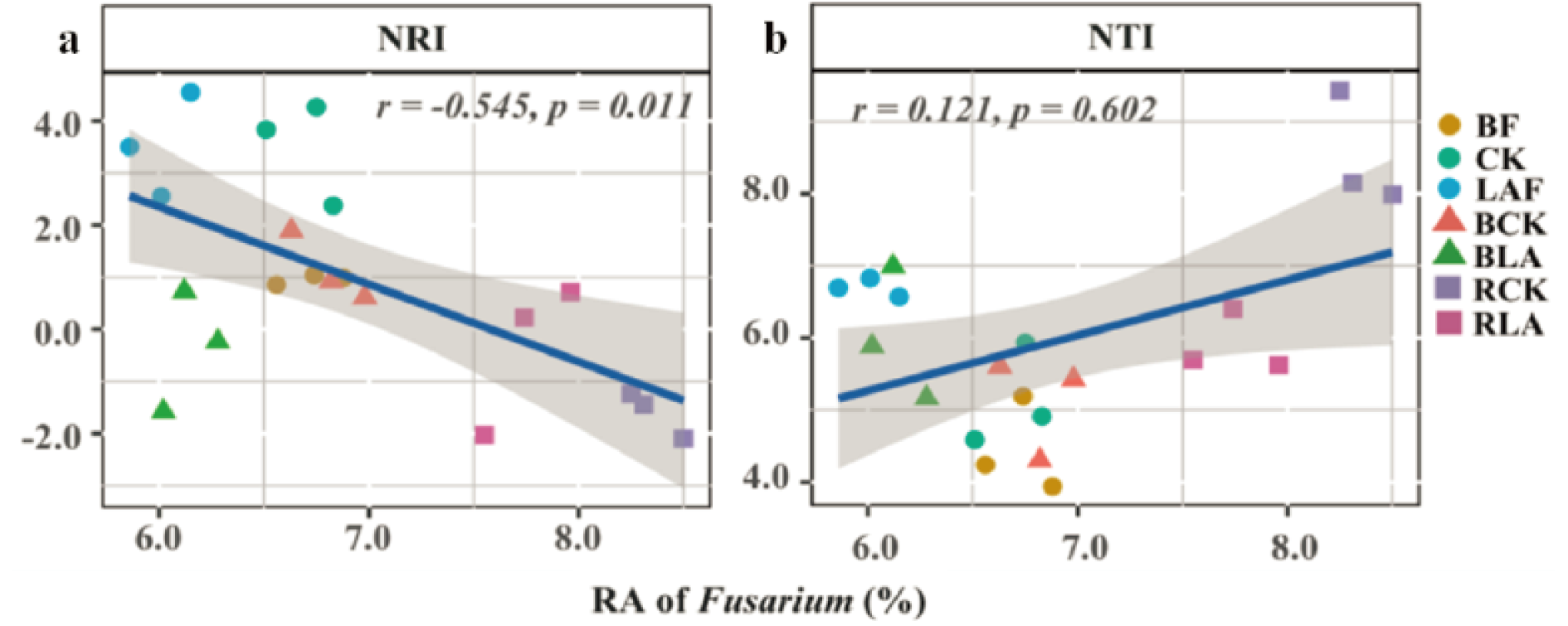

Supplement: Supplementary file 3 — Fig. S3. Spearman correlations between the abundance of F. oxysproum quantified by qPCR with the phylogenetic relatedness of based on nearest net relatedness (NRI) (a) and nearest taxon index (NTI) (b) in bacterial community. [file MBT2-12-515-s003.tif]

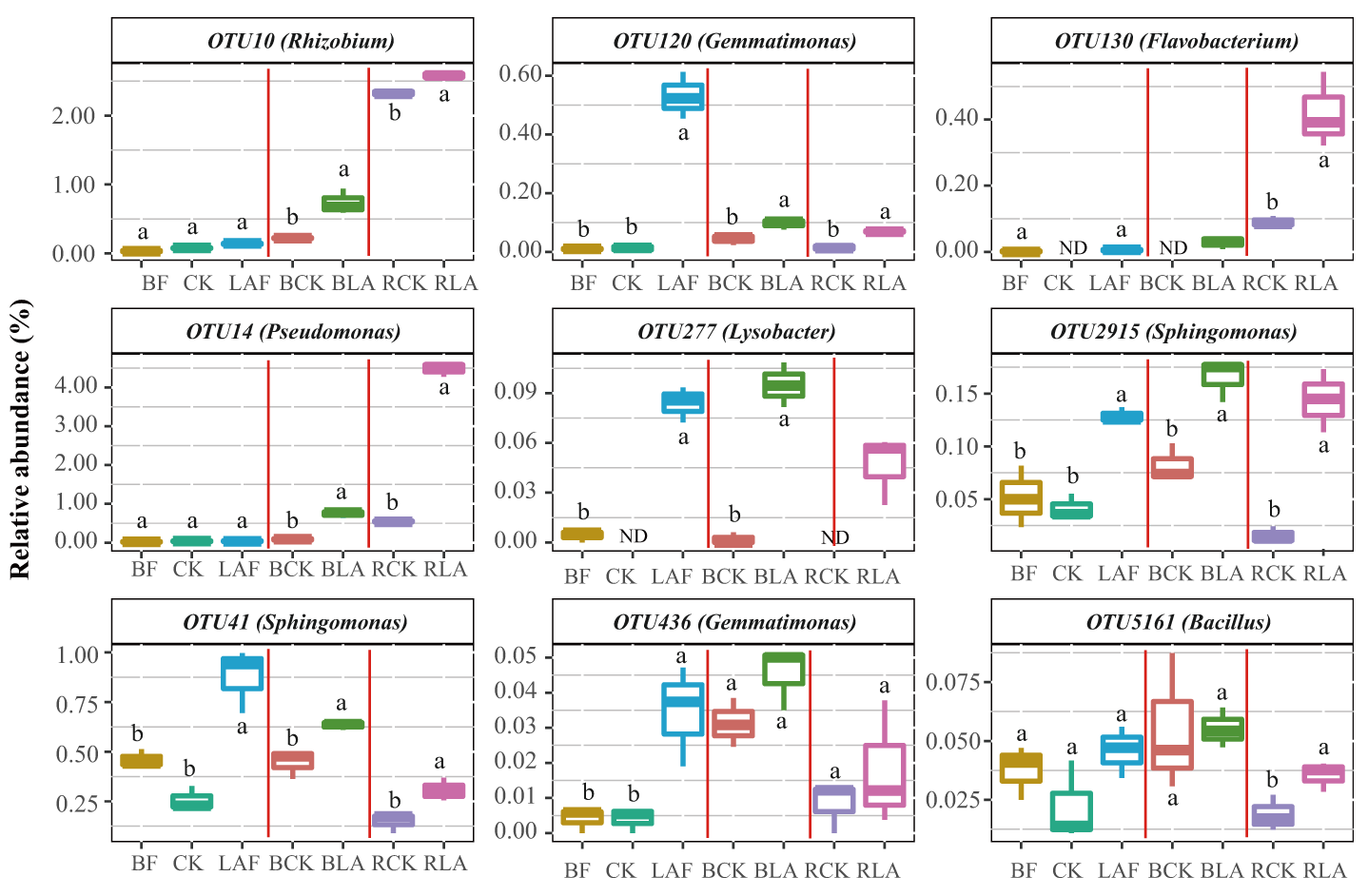

Supplement: Supplementary file 4 — Fig. S4. Boxplot of the relative abundance of OTU10 (Rhizobium), OTU120 (Gemmatimonas), OTU130 (Flavobacterium), OTU14 (Pseudomonas), OTU277 (Lysobacter), and OTU2915 (Sphingomonas) for each soil sample. [file MBT2-12-515-s004.tif]
