# Supplementary material for: Lime and ammonium carbonate fumigation coupled with bio‐organic fertilizer application steered banana rhizosphere to assemble a unique microbiome against Panama disease
Source: Microb Biotechnol. 2019 Mar 5;12(3):515–27. doi: 10.1111/1751-7915.13391 (PMC6465235; doi:10.1111/1751-7915.13391)
Supplement: Supplementary file 5 — Table S1. Number of sequences and OTUs from rawdata and processed final good quality sequences that were used to further analysis after basic quality control for different treatments. [file MBT2-12-515-s005.docx]

**Table S1** Number of sequences and OTUs from rawdata and processed final good quality sequences that were used to further analysis after basic quality control for different treatments.

| Sample | Rawdata | |  | Final | |
| --- | --- | --- | --- | --- | --- |
|  | No. of sequences | No. of OTUs |  | No. of sequences | No. of OTUs |
| BF1 | 19,286 | 3,179 |  | 16,999 | 2,787 |
| BF2 | 10,986 | 2,483 |  | 9,788 | 2,213 |
| BF3 | 17,668 | 3,095 |  | 16,007 | 2,725 |
| BCK1 | 14,543 | 2,596 |  | 13,001 | 2,330 |
| BCK2 | 21,679 | 3,107 |  | 19,457 | 2,620 |
| BCK3 | 18,210 | 2,899 |  | 16,269 | 2,551 |
| BLA1 | 18,852 | 2,293 |  | 17,133 | 2,034 |
| BLA2 | 28,063 | 2,650 |  | 25,349 | 2,213 |
| BLA3 | 26,859 | 2,651 |  | 23,956 | 2,218 |
| CK1 | 15,844 | 2,814 |  | 14,481 | 2,472 |
| CK2 | 20,462 | 3,067 |  | 18,451 | 2,523 |
| CK3 | 18,481 | 2,880 |  | 16,794 | 2,531 |
| LAF1 | 24,574 | 2,835 |  | 23,332 | 2,411 |
| LAF2 | 27,449 | 2,851 |  | 26,248 | 2,460 |
| LAF3 | 16,807 | 2,412 |  | 16,055 | 2,147 |
| RCK1 | 11,298 | 1,384 |  | 11,059 | 1,275 |
| RCK2 | 24,103 | 2,007 |  | 23,570 | 1,793 |
| RCK3 | 16,704 | 1,670 |  | 16,404 | 1,521 |
| RLA1 | 27,281 | 2,114 |  | 26,577 | 1,860 |
| RLA2 | 25,449 | 2,034 |  | 24,816 | 1,783 |
| RLA3 | 10,809 | 1,375 |  | 10,577 | 1,259 |
| Total | 415,407 | 7,580 |  | 386,323 | 5,448 |
