# Supplementary material for: Lime and ammonium carbonate fumigation coupled with bio‐organic fertilizer application steered banana rhizosphere to assemble a unique microbiome against Panama disease
Source: Microb Biotechnol. 2019 Mar 5;12(3):515–27. doi: 10.1111/1751-7915.13391 (PMC6465235; doi:10.1111/1751-7915.13391)
Supplement: Supplementary file 6 — Table S2. Spearman correlation coefficients and significant p value between the relative abundance of F. oxysproum with abundant bacterial and fungal phyla. [file MBT2-12-515-s006.docx]

**Table S2** Spearman correlation coefficients and significant p value between the relative abundance of *F. oxysproum* with abundant bacterial and fungal phyla.

| Phyla | r | *p* value |
| --- | --- | --- |
| Acidobacteria | -0.374 | 0.095 |
| Actinobacteria | -0.640 | 0.002 |
| Bacteroidetes | 0.370 | 0.099 |
| Chloroflexi | -0.538 | 0.012 |
| Firmicutes | -0.596 | 0.004 |
| Gemmatimonadetes | -0.669 | <0.001 |
| Proteobacteria | 0.782 | <0.001 |
| Verrucomicrobia | 0.827 | <0.001 |
