# Supplementary material for: Lime and ammonium carbonate fumigation coupled with bio‐organic fertilizer application steered banana rhizosphere to assemble a unique microbiome against Panama disease
Source: Microb Biotechnol. 2019 Mar 5;12(3):515–27. doi: 10.1111/1751-7915.13391 (PMC6465235; doi:10.1111/1751-7915.13391)
Supplement: Supplementary file 7 [file MBT2-12-515-s007.pdf]

**Microbial Biotechnology**

## Electronic supplementary material

# Lime and ammonium carbonate fumigation coupled with bio-organic fertilizer application steered banana rhizosphere to assemble a unique microbiome against Panama disease

Zongzhuan Shen[1], Beibei Wang[2], Jiaxin Zhu[1], Hangwei Hu[3], Chengyuan Tao[1], Yannan Ou[1], Xuhui Deng[1], Ning Ling[1], Rong Li[1, 4, *], Qirong Shen[1]

[1] Jiangsu Provincial Key Lab of Solid Organic Waste Utilization, Jiangsu Collaborative Innovation Center of Solid Organic Wastes, Educational Ministry Engineering Center of Resource-saving fertilizers, Nanjing Agricultural University, Nanjing 210095, Jiangsu, P. R. China

[2] Hainan key Laboratory for Sustainable Utilization of Tropical Bio-resources, Institute of Tropical Agriculture and Forestry, Hainan University, 570228 Haikou, P. R. China

[3] Faculty of Veterinary and Agricultural Sciences, University of Melbourne, Melbourne, Victoria 3010, Australia

[4] Ecology and Biodiversity Group, Department of Biology, Institute of Environmental Biology, Utrecht University, Utrecht, Netherlands

[*] Corresponding author: College of Resources and Environmental Sciences, Nanjing Agricultural University, 210095, Nanjing, China. E-mail: lirong@njau.edu.cn. Tel: (86)02584395535. Fax: (86)02584395535.

**Fig. S1** Sketch map for each season of the two seasonal greenhouse experiments.

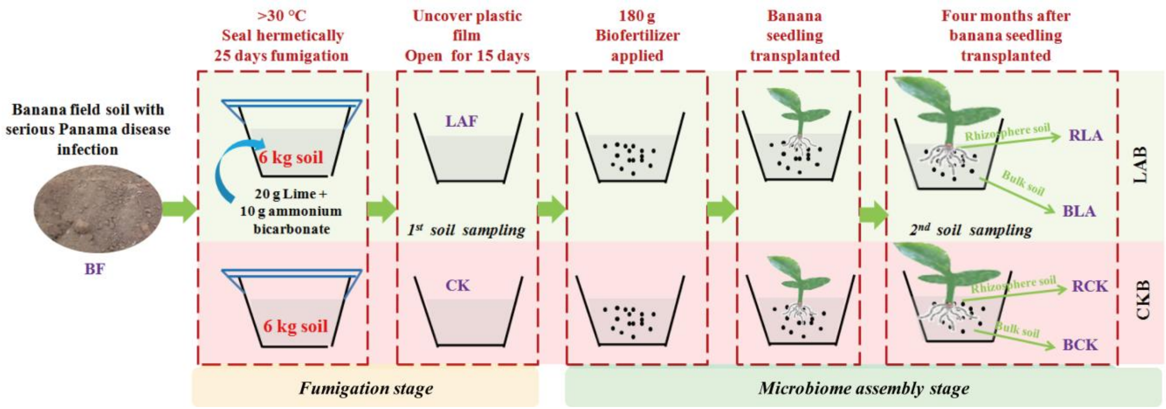

**Fig. S2** Heatmap displaying the abundance of dominated bacterial phyla in each soil sample. Numbers in each cell represent the mean relative abundance of each phylum.

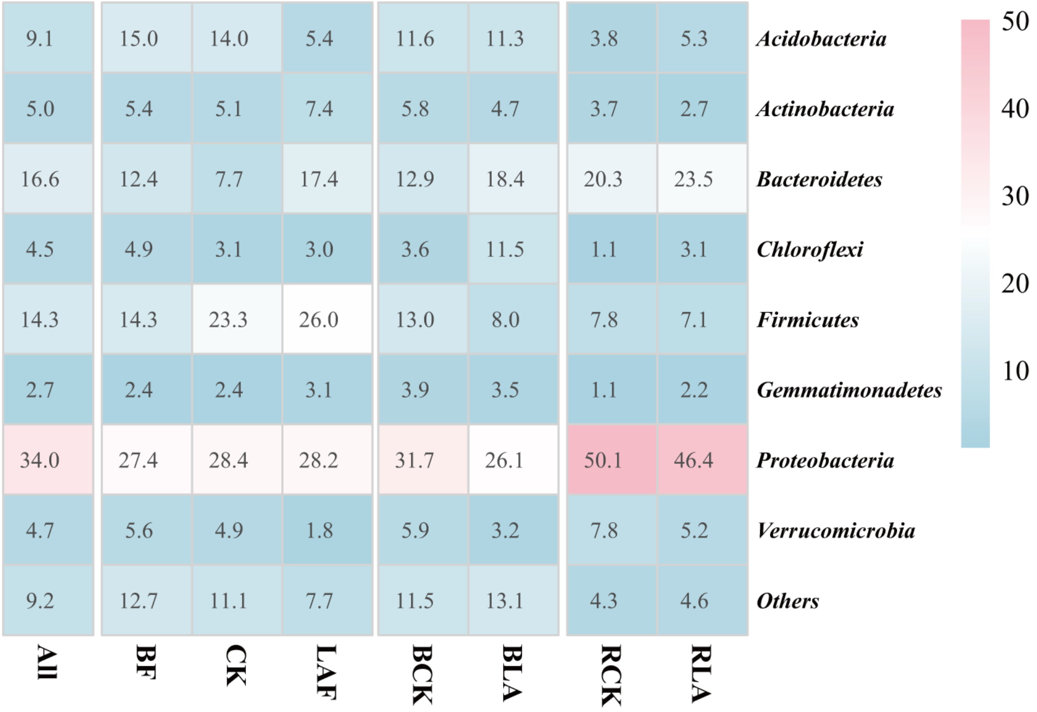

**Fig. S3** Spearman correlations between the abundance of *F. oxysproum* quantified by qPCR with the phylogenetic relatedness of based on nearest net relatedness (NRI) (a) and nearest taxon index (NTI) (b) in bacterial community.

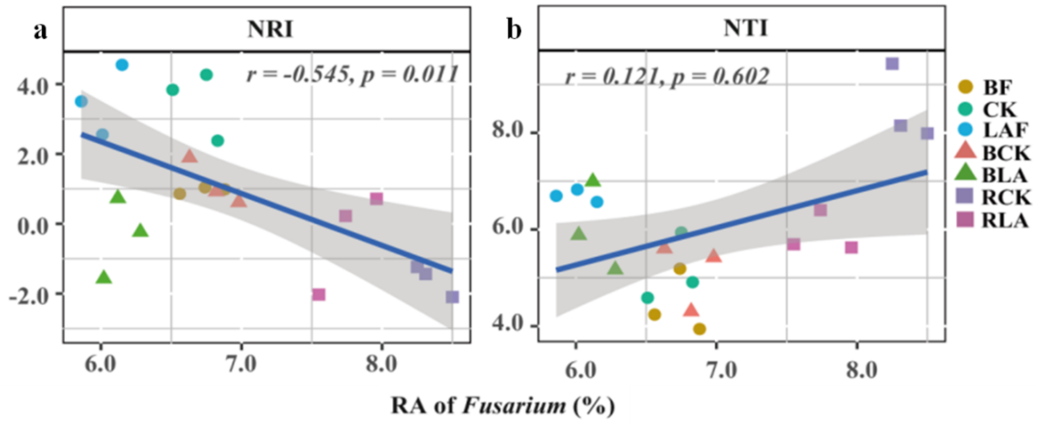

**Fig. S4** Boxplot of the relative abundance of OTU10 (*Rhizobium*), OTU120 (*Gemmatimonas*), OTU130 (*Flavobacterium*), OTU14 (*Pseudomonas*), OTU277 (*Lysobacter*), and OTU2915 (*Sphingomonas*) for each soil sample. Different letters indicate a significant difference ($p$ < 0.05) according to two independent sample t-test or ANOVA analysis.

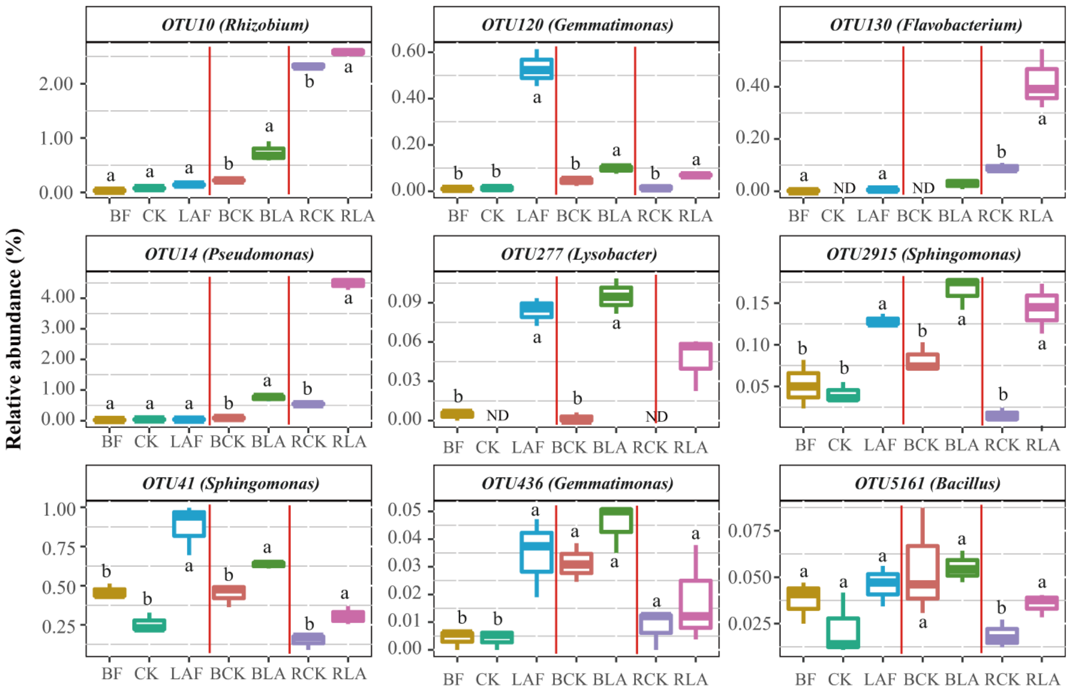

**Table S1** Number of sequences and OTUs from rawdata and processed final good quality sequences that were used to further analysis after basic quality control for different treatments.

| Sample | Rawdata | | Final | |
|---|---|---|---|---|
| | No. of sequences | No. of OTUs | No. of sequences | No. of OTUs |
| BF1 | 19,286 | 3,179 | 16,999 | 2,787 |
| BF2 | 10,986 | 2,483 | 9,788 | 2,213 |
| BF3 | 17,668 | 3,095 | 16,007 | 2,725 |
| BCK1 | 14,543 | 2,596 | 13,001 | 2,330 |
| BCK2 | 21,679 | 3,107 | 19,457 | 2,620 |
| BCK3 | 18,210 | 2,899 | 16,269 | 2,551 |
| BLA1 | 18,852 | 2,293 | 17,133 | 2,034 |
| BLA2 | 28,063 | 2,650 | 25,349 | 2,213 |
| BLA3 | 26,859 | 2,651 | 23,956 | 2,218 |
| CK1 | 15,844 | 2,814 | 14,481 | 2,472 |
| CK2 | 20,462 | 3,067 | 18,451 | 2,523 |
| CK3 | 18,481 | 2,880 | 16,794 | 2,531 |
| LAF1 | 24,574 | 2,835 | 23,332 | 2,411 |
| LAF2 | 27,449 | 2,851 | 26,248 | 2,460 |
| LAF3 | 16,807 | 2,412 | 16,055 | 2,147 |
| RCK1 | 11,298 | 1,384 | 11,059 | 1,275 |
| RCK2 | 24,103 | 2,007 | 23,570 | 1,793 |
| RCK3 | 16,704 | 1,670 | 16,404 | 1,521 |
| RLA1 | 27,281 | 2,114 | 26,577 | 1,860 |
| RLA2 | 25,449 | 2,034 | 24,816 | 1,783 |
| RLA3 | 10,809 | 1,375 | 10,577 | 1,259 |
| Total | 415,407 | 7,580 | 386,323 | 5,448 |

**Table S2** Spearman correlation coefficients and significant *p* value between the relative abundance of *F. oxysproum* with abundant bacterial and fungal phyla.

| Phyla | r | *p* value |
|---|---|---|
| Acidobacteria | -0.374 | 0.095 |
| Actinobacteria | -0.640 | 0.002 |
| Bacteroidetes | 0.370 | 0.099 |
| Chloroflexi | -0.538 | 0.012 |
| Firmicutes | -0.596 | 0.004 |
| Gemmatimonadetes | -0.669 | 0.001 |
| Proteobacteria | 0.782 | <0.001 |
| Verrucomicrobia | 0.827 | <0.001 |
